# Advances in Oocyte Maturation In Vivo and In Vitro in Mammals

**DOI:** 10.3390/ijms24109059

**Published:** 2023-05-21

**Authors:** Yao Jiang, Yingting He, Xiangchun Pan, Penghao Wang, Xiaolong Yuan, Bin Ma

**Affiliations:** 1 School of Medical, Molecular and Forensic Sciences, Murdoch University, Murdoch, WA 6149, Australia; 2 Shenzhen Branch, Guangdong Laboratory for Lingnan Modern Agriculture, Genome Analysis Laboratory of the Ministry of Agriculture, Agricultural Genomics Institute at Shenzhen, Chinese Academy of Agricultural Sciences, Shenzhen 518120, China; 3 Guangdong Laboratory of Lingnan Modern Agriculture, National Engineering Research Center for Breeding Swine Industry, Guangdong Provincial Key Laboratory of Agro-Animal Genomics and Molecular Breeding, College of Animal Science, South China Agricultural University, Guangzhou 510642, China

**Keywords:** folliculogenesis, oogenesis, oocyte, in vivo maturation, in vitro maturation, single-cell mRNA sequencing

## Abstract

The quality and maturation of an oocyte not only play decisive roles in fertilization and embryo success, but also have long-term impacts on the later growth and development of the fetus. Female fertility declines with age, reflecting a decline in oocyte quantity. However, the meiosis of oocytes involves a complex and orderly regulatory process whose mechanisms have not yet been fully elucidated. This review therefore mainly focuses on the regulation mechanism of oocyte maturation, including folliculogenesis, oogenesis, and the interactions between granulosa cells and oocytes, plus in vitro technology and nuclear/cytoplasm maturation in oocytes. Additionally, we have reviewed advances made in the single-cell mRNA sequencing technology related to oocyte maturation in order to improve our understanding of the mechanism of oocyte maturation and to provide a theoretical basis for subsequent research into oocyte maturation.

## 1. Introduction

Statistics show that the average maternal age at first birth has risen steadily in many industrialized countries since the 1980s. There are numerous reasons why women choose to delay childbearing, including work pressure, improved education, and mental/financial readiness to be good mothers [1,2]. However, female fertility declines with age, reflecting a decline in oocyte quantity and quality [3]. The quality of oocytes not only plays a decisive role in fertilization and embryo success, but also has a long-term effect on fetal development and growth in later stages [4].

Infertility, a disease/disorder of the male/female reproductive system causing failure to achieve a pregnancy after 12 months or more of regular unprotected sexual intercourse (or therapeutic donor insemination) affects up to 15% of couples worldwide [5]. Assisted reproductive technology (ART), a medical revolution that includes a range of techniques to improve fertility and achieve conception, is a boon for women unable to conceive at optimal reproductive age. Indeed, more than 5 million babies have been born through assisted conception worldwide, and the trend is increasing by more than 1 million yearly [6]. Currently, mice are widely used as models for human ART programs [7,8]. In addition, to meet the demand for meat as the world’s population increases, in vitro production (IVP) has become an attractive area of agricultural research, especially on livestock animals such as pigs [9], bovine [10], and sheep [11].

In vitro maturation (IVM) of the oocyte is a major part of the technology in the early stage of ART/IVP, and the analysis of oocyte maturation is a crucial step in the development of ART therapy [12]. Most studies have shown that successful IVM requires not only nuclear maturation, but also cytoplasmic maturation [13,14]. In vivo, oocyte maturation is regulated by numerous factors (e.g., follicle-stimulating hormone (FSH), luteinizing hormone (LH)) and the interaction between oocytes and granulosa cells [15,16,17]. In vitro, various medium formulations have been evaluated to create conditions more similar to those in vivo during oocyte maturation [18,19]. In the past few decades, improving oocyte maturation rates and oocyte quality in vitro has been the focus of many medical scientists. To date, considerable progress has been made in the recovery of oocyte maturation, the regulation/expression of related genes, and the stimulation of oocyte maturation by hormones. 

Although the optimization of hormone levels, molecular nutrition, and medium quality has significantly improved the environment for IVM of oocytes, the quality of in vitro maturation oocytes remains different from that of in vivo oocytes [20], possibly for the following reasons: (a) the mechanism of the acquisition of the developmental capacity of oocyte maturation is still not fully understood; (b) differences exist between in vitro culture and the in vivo ecological niche; or (c) transcriptome and epigenetic modifications during oocyte development have not been fully resolved. Therefore, this review mainly describes the oocyte development and maturation process, the relationship between granulosa cells and oocytes, and related studies on the oocyte maturation transcriptome. On the one hand, it aims to improve the understanding of the oocyte maturation process. On the other hand, it provides some references for future studies by explaining the existing research methods for oocyte maturation. Furthermore, it gives a theoretical basis for follow-up research and clinical applications of oocyte maturation.

## 2. Development of Ovarian Follicles in Mammals

**Figure 1:**
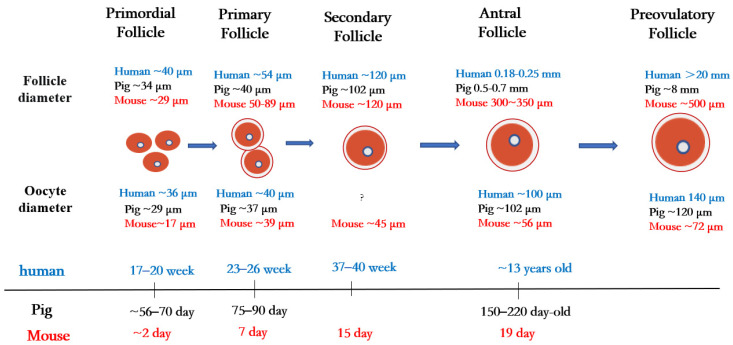
Folliculogenesis and oogenesis in humans, pigs, and mice.

In mammals, the ovary not only contains germ cells to produce offspring, but also controls numerous aspects of female development and physiology. The follicle is the basic functional unit of the ovary, and follicular development passes through stages termed primordial follicles, primary follicles, secondary follicles, antral follicles, and preovulatory follicles (Figure 1). 

Primordial follicles are the first type of follicles formed in mammalian ovaries. In humans, germ cells and granulosa cells begin to form primordial follicles at 17–20 weeks of fetal life, the size of the primordial follicles being about 40 µm in diameter [21]. In pigs, primordial follicles appear at 56 to 70 days of embryonic life, with follicle size being about 34 µm in diameter [22]. In mice, oocytes are fully assembled into the primordial follicular reserve only after birth, usually within 2–3 days after birth [23], with a follicle size of about 29 µm in diameter [24] and an oocyte size of about 17 µm in diameter [25]. 

Once granulosa cells proliferate and oocytes grow larger, they begin to secrete a series of glycoproteins assembled into a casing called the zona pellucida, which surrounds the oocyte and separates it from the granulosa cells. During the transition from the primordial follicle to the primary follicle, the oocyte genome is activated, and oocytes now secrete many factors of the transforming growth factor β (TGF-β) family. Growth differentiation factor 9 (GDF9), bone morphogenetic protein 15 (BMP15), and basic fibroblast growth factor (bFGF) produced by oocytes regulate primitive follicular growth [26]. At 23–26 weeks in human embryos and 75–90 days in pig embryos, the primordial follicle is activated, and the flattened granulosa cells grow into fusiform cubic cells surrounding the oocyte [27]. Follicles at this stage are called primary follicles. The diameter of the primary follicle is about 54 µm in the human ovary, 40 µm in the pig ovary [21], and 50–89 μm in the mouse ovary [28]. After primary follicle formation, most follicles will gradually be directed to apoptosis and atresia [29]. Later, the monolayer granulosa cells of some follicle cells proliferate into multiple layers, and these follicles begin to acquire their own vascular system and to form secondary follicles around the oocyte.

During the subsequent stages of folliculogenesis, small, fluid-filled cavities are formed within the follicle, ultimately fusing together to form a larger single cavity (known as the antrum), thus forming the antral follicle [30]. The granulosa cells in early antral follicles are classified into two groups: the cumulus granulosa cells, which are adjacent to the oocyte, and the mural granulosa cells, which line the follicle wall and serve as the primary source for steroid hormones [31]. In humans, early antral follicles appear during puberty (at about 13 years old), whereas in pigs, they typically occur between 150 and 220 days of age [32]. 

In cattle, scientists have compared and evaluated the developmental capacity of small follicles (≤4 mm in diameter), medium follicles (4–6 mm in diameter), and large follicles (≥6 mm in diameter) during in vitro maturation/fertilization and found that oocytes in large follicles have a higher developmental capacity than oocytes in the other two types of follicles (medium and small) [33]. This study suggests that the diameter of the primary oocyte is proportional to the diameter of the follicle and is related to its mature state and ability to support further embryonic development.

## 3. Maturation Process of Oocytes in Mammals

The oocyte, one of the largest cells in the mammalian body, is a female gametocyte (or germ cell) involved in reproduction. The maturation of oocytes has four important stages, namely, the germinal vesicle (GV) stage, germinal vesicle breakdown (GVBD), metaphase I (MI), and metaphase II (MII) [13,34]. In the ovary, the oocyte is arrested during the first meiotic prophase, also known as the GV stage. During the GV stage, the oogonium becomes a primary oocyte, passing through leptotene, zygotene, and pachytene to the diplotene stage. During late diplonema, the oocyte nucleus develops significantly, its chromatin becomes highly loosened, and the nucleus is then called a GV [35,36].

During oogenesis, oocytes regulate their gene expression by changing their chromatin structure. At the GV stage, the degree of chromatin aggregation and the spatial distribution of oocytes change, resulting in various configurations. In guinea pigs, chromatin structure has been classified into three configurations according to the density of the chromatin and whether the nucleolus is surrounded by chromatin. The three configurations include non-surrounded nucleoli (NSN; chromatin is scattered throughout the nucleolus region), surrounded nucleoli-1 (SN-1; some of the chromatin condenses around the nucleolus), and surrounded nucleoli-2 (SN-2; all chromatin is concentrated around the nucleolus). Our studies have shown that SN-2 oocytes have the potential to mature [37]. 

During porcine oocyte maturation [38], according to the chromatin configuration, the GV oocyte can be classified into two types, namely, the SN type (the chromatin forms a ring around the nucleoli) and the NSN type (chromatin spreads around the nucleoli). The nuclei of most oocytes close to ovulation show the SN karyotype configuration. Similar features have been observed in mice [39,40]. 

Other configuration classifications of oocytes at the GV stage have also been observed in other species. For example, in dairy cows, GV can be divided into four stages: GV0, GV1, GV2, and GV3. The GV0 phase has a diffuse filamentous chromatin pattern throughout the nuclear region. GV1 shows almost no foci of chromatin agglutination in the nucleus. GV2 exhibits chromatin condensed into various clumps or chains, whereas in GV3, the chromatin is condensed into single clumps within the nuclear envelope [41]. In goat oocytes, GV chromatin can be classified into GV1 (characterized by large nucleoli and diffuse chromatin) and GV2 (characterized by medium nucleoli and agglomerated network (GV2n) or clumping (GV2c) according to nucleolar size and the degree of chromatin aggregation). GV3 oocytes have small nucleoli and reticulated (GV3n) or lumped (GV3c) chromatin, whereas GV4 oocytes have no nucleoli but lumped chromatin. The functional differentiation between GV3n and GV3c has been suggested as a reference for the SN configuration of other species [42].

In dogs, the GV chromatin of oocytes is classified into four configurations (GV-I, -II, -III, and -IV) according to the degree of chromatin separation and concentration. The majority (86.7%) of ovulating oocytes in vivo have been shown to be in the GV-IV stage [43]. Moreover, the transcriptional activity of GV oocytes with two different chromatin configurations has been analyzed, and oocytes with NSN chromatin configurations have been shown to have stronger transcriptional activities than those with SN configurations. Nevertheless, transcription levels are lower in oocytes with the SN chromatin configuration [38,44], suggesting that the chromatin configuration at GV is related to developmental ability. The mechanism of transcriptional activity inhibition in GV phase endows oocytes with sufficient developmental potential and meets the molecular accumulation/metabolic requirements necessary for the subsequent growth and development of oocytes.

In mammals, GV oocyte arrest usually lasts several years as the oocyte waits for a signal to re-enter meiosis. Early studies confirmed that maintaining high levels of cyclic adenosine monophosphate (cAMP)in oocytes was required for meiotic arrest. Recent results using knockout mice or microinjection of inhibitory factors have shown that oocytes can produce sufficient cAMP to maintain meiotic arrest by activating adenylyl cyclase via the G protein-coupled receptor 3 (GPCR3) and/or G protein-coupled receptor 12 (GPR12) [45].

In ovarian granulosa cells, C-natriuretic peptide (CNP) and its receptor guanylyl cyclase, natriuretic peptide receptor 2 (NPR2), are key regulators of cyclic guanosine monophosphate (cGMP) homeostasis. When the LH level of the physiological cycle is increased, CNP/NPR2 complex activity decreases, which leads to a decrease in the cGMP level in granulosa cells and oocytes. The concentration of cAMP in oocytes is related to phosphodiesterase 3A (PDE3A) degradation. A decrease in the cGMP concentration can increase the hydrolytic activity of PDE3A, thereby reducing the cAMP level in oocytes. This leads to maturation-promoting factor (MPF) activation and resumption of meiosis [13]. In in vitro cultured oocytes, the discharge of the first polar body (PB1) is generally regarded as an indication of the nuclear maturation of oocytes.

Spindle assembly is essential to ensure proper separation of chromosomes in oocytes. In mitotic somatic cells, the centrosome is the main center of microtubule organization. However, many oocytes do not have a typical centrosome structure [46]. In mouse oocytes, acentriolar microtubule-organizing centers (aMTOCs) are responsible for meiosis spindle assembly. After meiosis recovery, the fragmented MTOCs merge into two focal points on opposite sides of the chromosome cluster and begin to form bipolar spindles [47]. The human oocyte microtubule-organizing center (huoMTOC) is the main structure of human oocyte microtubule assembly. A single huoMTOC forms in the cortex of the human GV oocyte and migrates to the nuclear membrane before nuclear membrane rupture (NEBD). After NEBD, huoMTOC is recruited to the centromere to initiate spindle microtubule nucleation. Studies have shown that centriolar coiled-coil protein 110 (CCP110), cytoskeleton-associated protein 5 (CKAP5), disrupted in schizophrenia 1 (DISC1), and transforming acidic coiled-coil containing protein 3 (TACC3) are essential for oocyte microtubule polymerization and spindle assembly. The absence of these proteins can lead to a structural breakdown of the huoMTOC, resulting in defects in spindle assembly and oocyte maturation [48]. 

Actin plays a vital role in the asymmetric division of oocytes. After GVBD rupture, microtubules form a spindle in the prokaryotic tissue. At this time, actin is evenly distributed in the oocyte, and the spindle moves roughly along its axis toward the cortex until the anterior pole of the spindle reaches the oocyte cortex at the MI end. This is followed by chromosome separation and the expulsion of PB1 from the oocyte. After the first division, the oocyte rapidly enters the metaphase of meiosis II (MII), in which the spindle forms below the cortex and maintains its asymmetric position during metaphase II stasis. The cortical myosin II ring also forms above the spindle. When the sperm penetrates the oocyte, the spindle rotates, and the oocyte extrudes the second polar body (PB2) [49,50]. Indeed, the oocyte does not complete meiosis until fertilization occurs [51].

## 4. Oocyte Nuclear Maturation and Cytoplasm Maturation

The capacity for fertilization and development is acquired by the oocyte after a lengthy period of growth and development from the embryonic stage to puberty [52,53,54,55]. The maturation of the oocytes includes two aspects: nuclear maturation and cytoplasmic maturation. The nuclear maturation of oocytes mainly refers to the separation of chromosomes, which reflects the ability of oocytes to resume meiosis. The nuclear maturation of the maturing oocyte accompanies the whole follicular development phase. 

In pigs, the synthesis of RNAs (up to 60–70% are ribosomal RNAs (rRNAs)) increases during oocyte growth and reaches a peak when the formation of the follicular antrum starts [56], suggesting that nuclear maturation occurs during the primordial, primary, and secondary stages in pigs. Typically, follicles larger than 0.8 mm in diameter indicate the beginning of follicular antrum formation in pigs [52]. 

Follicle development is accompanied by primordial follicle, primary follicle, and secondary follicle stages. By the GV stage, the nucleus reaches maturity. In pigs, the diameter of the follicle from the secondary follicle to the antral follicle is less than 3 mm, and the oocyte at this point is usually called a growing oocyte and does not resume meiosis in vitro. Oocytes are fully meiotic competent when their diameter is ≥3 mm, i.e., when these oocytes have reached cytoplasmic maturity.

Most (about 88%) growing porcine oocytes (approximately 100 μm in diameter) fail to mature in vitro. However, when nucleoli from fully grown (120 μm in diameter) porcine oocytes are re-injected into enucleated growing oocytes, 49% undergo maturation. Furthermore, the enucleated full-grown oocytes injected with nucleoli from fully grown oocytes mature to MII (56%), whereas injection with the nucleoli of growing oocytes reduces this maturation rate to 21% [57]; in this study, growing oocytes represent nuclear maturation, while fully grown oocytes represent cytoplasmic maturation. These results demonstrate that both nuclear maturation and cytoplasmic maturation are critical for oocyte maturation.

The cytoplasmic maturation of the oocytes involves the accumulation of mRNAs, proteins, and substrates required for subsequent fertilization/development [58]. As follicle growth and oocyte meiosis resume, transcription in the oocyte stops because of chromatin agglutination, and the maternal mRNAs stored in the oocyte are degraded and gradually consumed. Cytoplasmic polyadenylation of the 3′-untranslated region (3′-UTR) of mRNAs is strongly associated with mRNA stability and the activation of mRNA translation. Cytoplasmic polyadenylation element-binding protein 4 (CPEB4), which is associated with cytoplasmic polyadenylation element (CPE) located in the 3′-UTR of a specific mRNA, assembles an activator complex that promotes the translation of the target mRNA through cytoplasmic polyadenylation [59,60]. In addition, the maturation of organelles (e.g., mitochondria, endoplasmic reticulum) and cytoskeleton production/distribution are essential for oocyte maturation. In immature mouse oocytes, mitochondria accumulate around the GV and move away from the perinuclear region during GVBD. Mitochondria in MI and MII oocytes become more numerous and dispersed within the cytoplasm of the oocyte [61]. Mitochondria are the main sites of adenosine 5′-triphosphate (ATP) generation and provide the energy supply for oocytes. Evidence has been presented that reduced ATP levels lead to meiosis errors, which might alter the number of chromosomes in cells and lead to genetic disorders/diseases [62].

The endoplasmic reticulum is essential in protein degradation/ lipid metabolism and is the main reservoir for calcium ions. In GV-stage oocytes, the endoplasmic reticulum is evenly distributed within the cytoplasm of the whole oocyte. However, at MII, the endoplasmic reticulum is located in the cortical regions and aggregates in small clusters (1–2 µm) throughout the cytoplasm [63]. [Ca^2+^]i oscillations are crucial for triggering meiosis resumption/completion and oocyte maturation and early embryonic development [64]. Activation of the calcium signaling pathway in the endoplasmic reticulum is mediated by the inositol triphosphate receptor (IP3R). The number of IP3R1 increases during oocyte maturation, causing oocytes to produce persistent [Ca^2+^]i oscillations [65]. Increased cytoplasmic Ca^2+^ levels can also trigger the activity of calcium/calmodulin-dependent protein kinase II (CaMK II), which influences oocyte maturation. In addition, Ca^2+^ release can mediate the meiosis process in conjunction with ATP [66].

The maturation of the nucleus and cytoplasm of oocytes in vivo is synchronized. In vitro, however, when oocytes and granulosa cells are removed from follicles, meiosis spontaneously resumes, mainly because of the decreased cAMP concentration, resulting in desynchrony between cytoplasmic/nuclear maturation and reduced oocyte developmental competence. 

At present, simultaneous nuclear and cytoplasmic maturation is considered critical for obtaining high-quality oocytes in vitro [67]. For example, the addition of extracellular matrix (ECM) proteins to in vitro maturation media increases the oocyte maturation rate by increasing the levels of cytoplasmic maturation indicators (e.g., BMP15 and ATP) [68]. In addition, melatonin supplementation can also improve the cytoplasmic maturation of bovine oocytes significantly by improving the normal distribution of organelles and enhancing the expression levels of antioxidant genes (e.g., catalase, superoxide dismutase 1, and glutathione peroxidase 3) [69]. Moreover, the addition of cAMP modulators in the media can delay the nuclear maturation of oocytes, thereby achieving synchronous nucleo-cytoplasmic maturation and improving the quality of oocytes [70]. Therefore, our understanding of the factors that regulate oocyte development ability is gradually improved, and these insights have been slowly translated into incremental efficiency improvements in oocyte IVM.

## 5. Granulosa Cells Support Maturation of Oocytes

**Figure 2:**
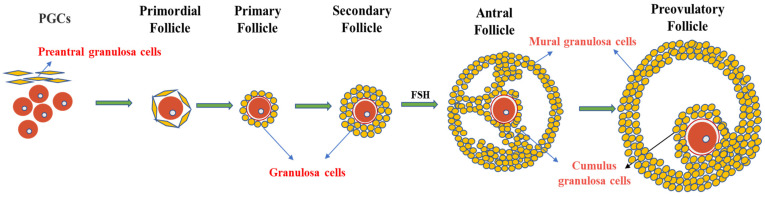
Granulosa cells and follicle development. Primordial follicles begin as oocytes, which are surrounded by a single layer of flattened cells called preantral granulosa cells. As the follicle develops, the flat granulosa cells become cubic and surround the oocyte in the primary follicle, accompanied by the appearance of a zona pellucida. In secondary follicles, one layer of granulosa cells multiplies into multiple layers. In the antral follicular stage, granulosa cells differentiate into mural granulosa cells and cumulus granulosa cells under the action of FSH.

The ovary contains follicles of various sizes. The smallest follicle, named the primordial follicle, consists of a single layer of preantral granulosa cells surrounding the oocyte. The largest follicle, named the Graafian follicle, consists of about 2–3 layers of cumulus granulosa cells and 5–10 layers of mural granulosa cells. Cumulus granulosa cells are the adjacent somatic cells of the oocyte, and their status and function have an important impact on the function of the oocyte (Figure 2). The major signaling pathways between oocytes and granulosa cells involve a paracrine pathway through specialized mesenchymal junctions called transzonal processes (TZP) [17]. TZP, a type of filamentous pseudopodium formed by the granulosa cells, can effectively achieve the exchange of metabolites and signaling molecules between granulosa cells and oocytes, including cyclic nucleotides (cAMP and cGMP) and some energy sources for oocyte development (e.g., lactic acid and pyruvate). In addition, some RNAs related to oocyte maturation can be transferred between the oocytes and granulosa cells via TZP [71]. In mice, the disruption of the gap junctions between oocytes and granulosa cells can lead to follicular arrest and the cessation of oocyte growth, suggesting that gap junction communication is essential for oocyte growth and meiosis [72].

Oocyte maturation requires a large amount of energy and nutrients for completion. As oocytes cannot transport certain amino acids or perform glycolysis/cholesterol biosynthesis, the maturation of oocytes requires metabolic synergy between oocytes and granulosa cells. During follicle development and oocyte maturation, the oocyte itself is dominant in this synergy [73]. Oocytes affect the expression of genes involved in glycolysis activity and the amino acid uptake of granulosa cells through paracrine signaling [15] and regulate the corresponding metabolic process in the granulosa cells. GDF9 and BMP15 are the two most important oocyte-secreted factors and function through BMP receptor type II. Oocytes influence cumulus granulosa cell proliferation [74], differentiation [75], and development [76] through paracrine pathways. Furthermore, oocytes can prevent the apoptosis of cumulus granulosa cells by maintaining the concentration of BMP15 [77]. When follicles are cultured in vitro, the relative levels of cumulus promoters are increased by supplementing GDF9 and BMP15 in order to regulate oocyte development [78]. The co-culture of oocytes with cumulus granulosa cells can restore FSH to stimulate the production of hyaluronic acid by cumulus granulosa cells, an indispensable step in the expansion of cumulus granulosa cells [79,80]. These results suggest that oocytes can affect the function of cumulus granulosa cells through oocyte-secreted factors.

Granulosa cells are essential in maintaining oocyte meiotic arrest and in regulating oocyte growth/development. Previous studies have shown that the expression of some genes in granulosa cells plays a critical role in oocyte development (Table 1). In addition, some small molecules/compounds, such as cyclic nucleotides (e.g., cAMP and cGMP), are believed to be key molecules controlling the meiosis of mammalian oocytes. Cyclic nucleotides derived from follicular cells and germ cells are the primary molecules that maintain meiotic arrest in oocytes [81]. In granulosa cells, natriuretic peptide C (NPPC)/NPR2 signaling regulates cGMP levels and engages in maintaining meiotic arrest in oocytes [82]. In addition, the estradiol (E2)–estrogen receptor (ER) system in granulosa cells maintains meiotic arrest of oocytes by binding to the NPPC/NPR2 promoter region and regulating their expression [83,84].

Co-culture of oocytes with granulosa cells can improve oocyte quality when inducing oocyte maturation in vitro [16]. Oocytes in the co-culture system have similar gene expression and developmental competence to in vivo mature oocytes [85]. In addition, in vitro induction of granulosa cell senescence and apoptosis has been shown to result in ovarian follicular dysplasia and oocyte dysfunction, suggesting that granulosa cells play an indispensable role in oocyte development [86,87]. Efforts are currently being made to improve techniques promoting oocyte development by co-culturing oocytes with granulosa cells, including organoid cultures [88] and parthenogenesis [89]. Therefore, considering the influence of granulosa cells on oocytes, the co-culture system for in vitro maturation of oocytes has been preferred by more researchers.

**Table 1:** Key genes involved in functions of granulosa cells and oocyte maturation.

Species	Gene	Effects on Granulosa Cells	Action on Oocytes, Follicles, or Ovaries	References
**Mouse**	*UFL1*	Inhibits cell apoptosis.	Relieves premature ovarian failure.	[90]
**Human**	*BMP8A*	Inhibits luteinization of granulosa cells.	Promotes normal ovulation of follicles.	[91]
**Human**	*PTEN*	Promotes granulosa cell apoptosis.	Affects follicular atresia.	[92]
**Mouse**	*BRE*	Inhibits granulosa cell apoptosis.	Inhibits follicular atresia.	[93]
**Human**	*RAC1*	Promotes cell proliferation and inhibits granulosa cell apoptosis.	Inhibits premature ovarian failure.	[94]
**Cow**	*ERβ*	Inhibits granulosa cell proliferation and induces granulosa cell autophagy.		[95]
**Goat**	*CYP19A1*	Promotes cell proliferation.	Reduces the secretion of estrogen and progesterone.	[96]
**Goat**	*TIMP1*	Promotes cell proliferation.	Ovarian development (affecting E2 secretion).	[97]
**Sheep**	*SIRT2*	Promotes granulosa cell amplification.	Promotes oocyte maturation.	[98]
**Yak**	*PPP1R11*	Promotes granulosa cell proliferation and inhibits apoptosis.	Maintains follicle development.	[99]
**Mouse**	*PRMT5*	Prevents premature differentiation of granulosa cells.	Maintains healthy follicle development in mice.	[100]
**Human**	*ADAMTS1*	Maintains granulosa cell proliferation.	Maintains normal secretion of E2 and promotes oocyte maturation.	[101]
**Human**	*PATL2*	Decreased expression-induced apoptosis of granulosa cells.	Maintains normal development and maturation of oocytes.	[102]
**Human**	*LNK*	Promotes granulosa cell apoptosis.	Reduces oocyte maturation and participates in ovulation disorders.	[103]
**Buffalo**	*AQP8*	Inhibition of its expression can inhibit granulosa cell apoptosis.	Affects follicle development.	[104]
**Rat**	*Bmp8*	Promotes cumulus granulosa cell expansion.	Improves maturation and development of closed oocytes.	[105]
**Pig**	*SIRT6*	Maintains the expansion of cumulus granulosa cells.	Maintains oocyte meiosis.	[106]
**Cow**	*CPEB3*	Inhibition of its expression could reduce the apoptosis of cumulus granulosa cells.		[107]

## 6. In Vitro Technology Promotes the Study of Oocyte Maturation

Oocyte maturation in vitro is a critical step in ART and its development. Successful IVM involves the maturation of the nucleus, cytoplasm, and related molecular pathways. Previous studies have shown that not all oocytes can develop into embryos after IVM, and that the culture system of mature oocytes in vitro significantly impacts their development ability. Until now, numerous approaches have been developed to facilitate the in vitro maturation of mammalian oocytes.

Conventional in vitro maturation of human oocytes has a low rate because of the lack of an ovarian niche. Knowledge of the bidirectional regulation between granulosa cells and oocytes has improved oocyte maturation in vitro by using the co-culture of oocytes and granulosa cells [85]. Two-dimensional (2D) models are more often used in co-cultures consisting of oocytes and granulosa cells. In order to mimic the microenvironment of oocytes in the ovary more closely, corresponding changes have been made toward the development of three-dimensional (3D) oocyte cultures. During IVM of human oocytes, the oocytes and granulosa cells cultured in 3D systems with hyaluronan show better viability and increased mitogen-activated protein kinase activity compared with 2D culture models [108]. In addition, in human IVM, scientists [109] have simulated a 3D co-culture system of naked oocytes and granulosa cells in a barium alginate film and found that the maturation rate of oocytes in the 3D co-culture system is significantly higher than that in the control 2D culture group. A recent IVM study in pigs has also demonstrated that the in vitro maturation rates of oocytes in a 3D agarose matrix are significantly higher than those without the agarose matrix, and that both the BMP15 levels in the granulosa cells and the ATP levels in the oocytes increase [110]. A variety of 3D in vitro co-culture models have been established in recent years. This innovative approach of granulosa cell–oocyte complex culture in vitro opens up new perspectives for studying human oocyte IVM.

Currently, oocytes undergoing IVM cannot achieve complete cytoplasmic maturation, and the basal medium needs to be supplemented with numerous known nutrients to promote oocyte maturation and development during IVM. FSH is one of the critical components of IVM media [18]. In goats [111], cattle [18], and mice [112], the addition of FSH to the oocyte maturation system in vitro increases the oocyte maturation rate by promoting cumulus expansion and influencing the cAMP level. 

Appropriate concentrations of LH and other gonadotropins [113,114], oocyte growth factors (including GDF9 and BMP15) [115], and epidermal growth factors [116] can also influence oocyte and embryo quality in vitro. In addition, exogenous gonadotropins, such as pregnant mare serum gonadotropin (PMSG) and human chorionic gonadotropin (hCG), are often utilized to promote the ovulation of oocytes during in vitro studies of oocyte development [117]. In porcine and bovine IVM, follicular fluid is also used in culture media. Epidermal growth factor (EGF) in follicular fluid can effectively trigger cumulus cell expansion and promote maturation, although the components of follicular fluid are not fully understood [118,119].

Reactive oxygen species (ROS) are by-products of mitochondrial metabolism and are almost unavoidable during oocyte maturation. Oxidative stress results from the imbalance between the production and elimination of reactive oxygen species in the cell. During oocyte culture in vitro, the balance between the production and elimination of intracellular ROS is lost because of rapid temperature changes during oocyte collection and exposure to visible light, and this directly affects the meiosis process, cytoskeletal structure, and gene expression, thus affecting oocyte quality [120,121]. To avoid the harmful effects of excessive ROS, the current strategy is to use a variety of antioxidants during IVM. For example, when nobiletin (a natural antioxidant product; at 25–50 μM), was added to the bovine oocyte culture system in vitro, oocytes showed higher cortical particle mobility/mitochondrial activity and decreased ROS/glutathione content [122]. Melatonin, an essential antioxidant, also plays a significant role in oocyte maturation and in delaying ovarian senescence [123,124]. Oral administration of melatonin to aged mice was found to increase TZP significantly, maintain cumulus cell–oocyte communication, enhance oocyte ATP levels, and decrease the ROS levels, cell apoptosis, DNA damage, endoplasmic reticulum stress, and spindle/chromosome defects [125].

Resveratrol (RES) is a natural antioxidant with the potential to effectively improve oocyte quality, reduce ROS levels, and inhibit apoptosis by restoring spindle/chromosome structure. Studies have shown that RES can protect oocytes from Adriamycin-induced DNA damage [126]. In addition, coenzyme Q10 [70] can protect oocytes from ROS by improving mitochondrial function, thus enhancing the quality of oocytes cultured in vitro. Furthermore, tea polyphenols, as good antioxidants, can also reduce ROS accumulation during follicular formation, thus affecting oocyte maturation [69]. Therefore, an increasing number of antioxidants can be utilized as additives to improve the in vitro quality of oocytes.

In the past few decades, oocyte maturation in vitro has always been considered an experimental technique. However, since 2021, the American Society for Reproductive Medicine and other organizations have suggested that this technique is no longer experimental [127]. Overall, IVM can be considered a promising technology, especially for fertility-related clinical studies.

## 7. Single-Cell Multi-Omics Facilitates the Understanding of the Mechanisms of Oocyte Maturation

Over the past few decades, genome-wide analyses have revealed the molecular processes that drive development. The current focus is on the meiotic stages of oocytes from GV to MII. Studies of the core regulatory factors and signaling pathways of oocyte development during this period are of great significance for improving the in vitro maturation rate of oocytes. RNA sequencing (RNA-seq) can determine the sum of all transcribed RNAs in a tissue or cell under a given condition [128,129]. However, it cannot identify the genes causing differences between cells because samples for RNA-seq are usually obtained from a mixture of cells, an aspect that does not apply to precious cell types such as oocytes [130].

The development of single-cell omics techniques has provided new opportunities for studying the fine molecular regulation of oocyte maturation by capturing multiple omics layers from the same cell (e.g., genomics + transcriptomics, epigenomics + transcriptomics, and transcriptomics + targeted proteomics). Analysis of multiple omics layers can capture a complete set of information about each cell compared to any single omics layer, thereby better reflecting the complex network of interactions responsible for cellular function. An understanding of the genetic/regulatory changes in oocyte maturation and the gene communication between an oocyte and other cells in the follicle (by separating the nucleus/cytoplasm and analyzing the molecular events such as chromatin structure/modification and transcriptome) is critical for successful oocyte research.

The transcriptome changes undergone by human oocytes during IVM and the differential gene expression profile of each oocyte are now being revealed by using single-cell RNA sequencing (scRNA-seq) technology. The hypoxia-inducible factor 1-alpha and sirtuin signaling pathways might be the key players in the maturation of human oocytes in vitro [131]. In addition, scRNA-seq of goat GV and MII oocytes has identified 4516 differentially expressed genes (DEGs), including 16 histone methyltransferase and demethylase DEGs (including lysine-specific demethylase 1 (LSD1)). The positive effect of LSD1 on goat oocytes during IVM has been confirmed by adding LSD1 inhibitor (GSK-LSD1) into cultures undergoing oocyte maturation in vitro [132]. In another study, the gene expression patterns of GV and in vitro MII oocytes of donkeys have been investigated by scRNA-seq. A total of 24,164 oocyte-related genes have been identified, of which 9073 are significantly differentially expressed in GV and MII oocytes. Further analysis has shown that these genes participate in meiosis, mitochondrial activity, and N-glycan biosynthesis [133].

Studies of the molecular mechanisms of porcine meiosis by using the recently developed scRNA-seq technique to analyze the transcriptome of GV and MII oocytes have revealed that during the transition from GV to MII, a decrease occurs in the total number of RNAs (e.g., mRNAs, long noncoding RNAs (lncRNAs)) detected in oocytes. In addition, 1807 (602 up-regulated and 1205 down-regulated) mRNAs and 313 (177 up-regulated and 136 down-regulated) lncRNAs are significantly differentially expressed, indicating that more mRNAs are down-regulated, whereas more lncRNAs are up-regulated. Most importantly, mitochondrial mRNAs have been observed to be actively transcribed during the maturation of porcine oocytes [134]. Furthermore, in oocytes during the transition from human primordial follicles to primary follicles, 223 and 268 genes are down-regulated and up-regulated, respectively [135].

Furthermore, scRNA-seq can also reveal changes in gene expression in various cell types and organelles during oocyte maturation (Table 2). Granulosa cells are the close neighbors of oocytes, and their various gene expression patterns during oocyte meiosis are correlated with oocyte quality [136,137]. Single-cell transcriptome analysis of the follicular microenvironment surrounding MII oocytes in preovulation follicles of different individual females has been performed, and six different cell types, including granulosa cells/epithelial cells and various immune cells (e.g., macrophages, dendritic cells, T cells, and neutrophils) have been identified. Different functional clusters with different functional transcriptome profiles, including specific clusters involved in the inflammatory response and adhesion, have also been identified. Finally, the function of follicular macrophages in the immune response/ECM remodeling and the role of granulosa cells in promoting oocyte meiotic resumption have also been determined [138].

Moreover, scRNA-seq can also be utilized in the study of meiosis-related diseases. For example, the use of scRNA-seq data from healthy and polycystic ovary syndrome (PCOS) oocytes at various stages of their maturation (including GV, MI, and MII), combined with bioinformatics, has confirmed that mitochondrial function is related to the failure of oocyte development in PCOS patients [144]. 

The single-cell transcriptomic analysis of oocytes from women with ovarian endometriosis and from healthy women has revealed changes in key biological processes and molecular functions related to steroid metabolism, the response to oxidative stress, and the regulation of cell growth, all of which probably lead to a reduced oocyte mass in women with endometriosis [145]. At present, possible directions of scRNA-seq in studies of meiosis are to utilize modern technologies of scRNA-seq or to combine it with other technologies. For example, single-cell transcriptome analysis can be combined with genomics, epigenetics, and proteomics. This type of combinational technique is called the single-cell multi-omics technique [149].

Epigenetic processes are integral parts of oocyte nuclear/cytoplasmic maturation and influence the development after fertilization. Many post-translational modifications occur in the developing oocyte, including acetylation, ubiquitination, and the methylation of various proteins. Histone deacetylases (HDACs) are essential for the lysine acetylation of histones. Studies have shown that inhibition of HDAC3 in porcine oocytes leads to increased acetylation of α-tubulin during oocyte meiosis, thereby affecting microtubule stability during porcine oocyte meiosis [150]. In mice, HDAC11 promotes oocyte maturation by reducing α-tubulin acetylation [151]. Ubiquitin C-terminal hydrolases (UCHs) comprise a family of deubiquitinating enzymes. In mouse and rhesus monkey oocytes, inhibition of UCH activity causes meiotic defects and chromosome misalignment, indicating that deubiquitination is vital for maintaining the regular progress of oocyte maturation [152].

In addition, abnormal methylation of maternal genes can also affect post-fertilization development. Previous studies have shown that two-thirds of all methylcytosines occur in non-CG (CA, CT, and CC) sites in mouse GV oocytes. Compared with non-growing oocytes (with immature nucleoli), GV oocytes are over four times more methylated at non-CG sites, suggesting that non-CG methylation accumulates during oocyte growth. Furthermore, the rate of CG methylation is 2.3% in non-growing oocytes (increasing to 37.9% in GV oocytes). Similarly, the methylation rate of non-CG sites increases from 0.61% to 3.2% from non-growing oocytes to GV oocytes [153]. Furthermore, 5’-C-phosphate-G-3’(CpG) methylation in general exhibits a dynamic profile during oocyte growth. Of all CpGs, 0.5% are hypermethylated in day 5 oocytes (≥80% methylation; with immature nucleoli), 11.3% in GV oocytes, and 15.3% in MII oocytes [154]. In humans, CpGs with decreased methylation from early GV to MI stage are more concentrated in CpG islands, and non-CpG methylation undergoes a large-scale remodeling from MI to MII [155].

## 8. Conclusions

Oocyte meiosis involves a complex and orderly regulatory process whose mechanism has not been fully elucidated. Arrest and recovery of GV oocytes significantly impact later oocyte development and involve many regulated hormonal signaling pathways. The contribution of granulosa cells to oocyte maturation is also indispensable. Future improvements in culture media and conditions should significantly enhance the quality of oocytes cultured in vitro. The development of related technologies, including those based on single-cell transcriptome analysis, should facilitate our understanding of oogenesis/oocyte maturation and the development of innovative approaches to promote oocyte maturation in vivo and in vitro for ART.

## Figures and Tables

**Table 2:** Advances in the single-cell transcriptome of oocytes in mammals.

Species	Cell Type	Period	Phenotype	Related Genes	References
**Mouse**	5-month-old and 32-month-old oocytes	GV	Ovarian aging	*CYP19A1*	[139]
**Goat**	High- and low-yielding ovarian granulosa cells	Follicles with diameters > 7 mm, 3–7 mm, and 1–3 mm	Follicular development	*ASIP*, *INHA*, *HSD17B1*, *CYP11A1*	[136]
**Human**	Six elderly (around 42 years old) and three young (around 25 years old)	MII	Oocyte senescence	*UBE2C*, *UBC*, *CDC34*	[140]
**Mouse**	Follicular granulosa cells and membrane cells	Secondary follicles, presinus follicles, sinus follicles, ovulation follicles	Follicular development	*CYP11A1*	[141]
**Human**	Granulosa cells in follicles	MII	Oocyte maturation	*STAR*, *CYP11A1*, *HSD3B2*, *CYP5A*	[138]
**Pig**	Oocyte	GV, MII	Oocyte maturation	*DNMT1*, *UHRF2*, *PCNA*, *ARMC1*	[134]
**Donkey**	Oocyte	CV, MII	Oocyte maturation	*DPPA3*, *PTTG1*, *BTG4*, *KPNA7*, *RNF34*	[133]
**Goat**	Oocyte	GV, MII	Oocyte maturation	*GTF2H3*, *MRPL37*, *TMEM128*, *PFDN1*	[132]
**Human**	Oocytes and surrounding granulosa cells	Preantral follicle	Follicular development	*FGF9*, *ERBB4*, *NECTIN1*, *FGFR2*	[142]
**Human**	Oocyte	GV, MII	Oocyte maturation	*OOSP2*	[143]
**Mouse**	Oocytes and Pre-granular cells	Primordial follicles	Oocyte development	*WNT4*, *TGFB1*, *FOXO3*	[25]
**Human**	Oocyte	GV, MI, MII	Oocyte maturation	*COX6B1*, *COX8A*, *COX4l1*, *NDUFB9*	[144]
**Human**	Oocyte (OE/health)	MII	Oocyte development	*APOE*, *DUSP1*, *G0S2*, *H2AFZ*, *PXK*	[145]
**Human**	Oocyte (two age groups)	GV, MII	Oocyte senescence	*SFXN5*, *CDA*, *SLC35A1*, *NPL*, *FBXO32*	[146]
**Human**	Oocytes and granulosa cells	Primordial, primary, secondary, antral, preovulatory	Follicular development	*BMPR2*, *HDC*, *HRH2*, *TNFSF13*, *CDC25C*, *NPM2*	[147]
**Human**	Oocyte (around 27 and 43)	MII	Oocyte maturation and ovarian senescence	*TOP2B*	[148]

## Data Availability

Not applicable.

## Abbreviations

2D	Two-dimensional
3D	Three-dimensional
3′-UTR	3′-untranslated region
aMTOCs	Acentriolar microtubule-organizing centers
ART	Assisted reproductive technology
ATP	adenosine 5′-triphosphate
bFGF	Basic fibroblast growth factor
BMP15	Bone morphogenetic protein 15
cAMP	Cyclic adenosine monophosphate
CaMK II	calcium/calmodulin-dependent protein kinase II
CNP	C-natriuretic peptide
cGMP	Cyclic guanosine monophosphate
CCP110	Centriolar coiled-coil protein 110
CKAP5	Cytoskeleton-associated protein 5
CPE	Cytoplasmic polyadenylation element
CPEB4	Cytoplasmic polyadenylation element-binding protein 4
CpG	5-C-phosphate-G-3
DEGs	Differentially expressed genes
DISC1	Disrupted in schizophrenia 1
E2	Estradiol
ECM	Extracellular matrix
EGF	Epidermal growth factor
ER	Estrogen receptor
FSH	Follicle-stimulating hormone
GDF9	Growth differentiation factor 9
GPCR3	G protein-coupled receptor 3
GPR12	G protein-coupled receptor 12
GV	Germinal vesicle
GVBD	Germinal vesicle breakdown
hCG	Human chorionic gonadotropin
HDACs	Histone deacetylases
huoMTOC	Human oocyte microtubule-organizing center
IP3R	Inositol triphosphate receptor
IVM	In vitro maturation
IVP	In vitro production
LH	Luteinizing hormone
LncRNAs	Long noncoding RNAs
LSD1	Lysine-specific demethylase 1
MI	Metaphase I
MII	Metaphase II
MPF	Maturation-promoting factor
NEBD	Nuclear envelope breakdown
NPPC	Natriuretic peptide C
NPR2	Natriuretic peptide receptor 2
NSN	Non-surrounded nucleoli
PB1	First polar body
PB2	Second polar body
PCOS	Polycystic ovary syndrome
PDE3A	Phosphodiesterase 3A
PMSG	Pregnant mare serum gonadotropin
rRNAs	Ribosomal RNAs
RNA-seq	RNA sequencing
ROS	Reactive oxygen species
scRNA-seq	Single-cell RNA sequencing
SN	Surrounded nucleoli
TACC3	Transforming acidic coiled-coil containing protein 3
TGF-β	Transforming growth factor β
TZP	Transzonal processes
UCHs	Ubiquitin C-terminal hydrolases
